# Intact cell MALDI mass spectrometry biotyping for "at-line" monitoring of apoptosis progression in CHO cell cultures

**DOI:** 10.1186/1753-6561-7-S6-P8

**Published:** 2013-12-04

**Authors:** Sebastian Schwamb, Bogdan Munteanu, Björn Meyer, Carsten Hopf, Mathias Hafner, Philipp Wiedemann

**Affiliations:** 1 Center for Applied Biomedical Mass Spectrometry (ABIMAS), Mannheim, Baden-Württemberg, 68163, Germany; 2 Mannheim University of Applied Sciences, Mannheim, Baden-Württemberg, 68163, Germany; 3 Heidelberg University, Institute for Medical Technology, Mannheim, Baden-Württemberg, 68163, Germany

## Background

Mammalian cell cultures, especially Chinese Hamster Ovary (CHO), are the predominant host for the production of biologics. Despite considerable progress in industry and academia alike (also enforced e.g. by the Process Analytical Technology Initiative of the FDA), particularly in the field of process monitoring there is still a need for innovative methods enabling improvement of process monitoring. For optimized process control it would be imperative to know as early as possible "when a cell needs what", when it is stressed, running into substrate limitations etc., at best in an online or robust at line format.

Intact cell MALDI mass spectrometry (ICM-MS) biotyping, a method used successfully in the field of clinical and environmental microbiology, is getting more attention in the context of mammalian cell cultivation. Here we report preliminary results of an assessment of a fast and high throughput at line capable ICM MS method for cell culture monitoring. As a first example, we choose apoptosis monitoring.

The identification of specific mass spectrometric signatures related to early stages of apoptosis using ICM-MS biotyping as reported here could be a promising tool for CHO culture.

## Material and methods

An exponentially growing CHO suspension cell line was inoculated at a seeding density of 2 × 10^5 ^cells/ml and an initial volume of 30 ml in 125 ml Erlenmeyer flasks. Samples for assessing viability- and apoptosis-progression and for ICM MS biotyping were taken at 48, 72, 96, 120, 144, 192 and 240 h. Experiments were carried out as biological triplicates.

Viability was determined by trypan blue dye exclusion using a ViCell (Beckman Coulter, Krefeld, Germany) for automated processing. Apoptosis was measured in triplicate for each biological sample by means of caspase-9 activity (Caspase-Glo^®^9 assay kit; Promega, Mannheim, Germany) using a microplate format (plate reader POLARstar Omega, BMG Labtech, Ortenberg, Germany).

ICM MS biotyping (using a Bruker Autoflex III MALDI-TOF/TOF MS) analysis samples were prepared from as little as 2500 cells. The method is described in detail by Munteanu et al. (2012) [1].

## Results

To evaluate the power of ICM MS as an at-line analytical method for apoptosis monitoring, batch cultivations of CHO suspension cells were analyzed by standard analytical methods and ICM MS in comparison.

**Figure 1:**
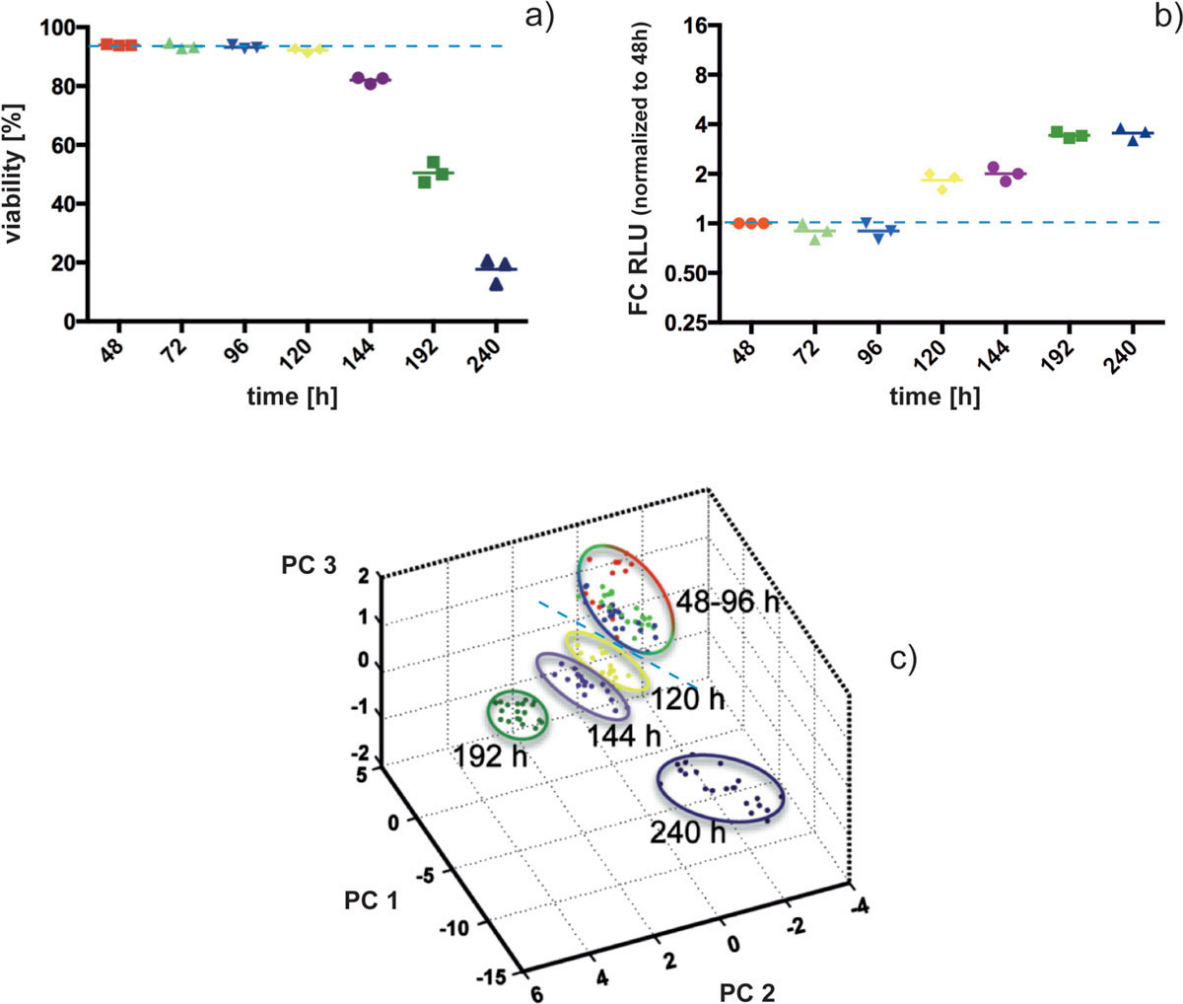
**Viability (a), caspase-9 activity (b) and ICM MS biotyping (c) during batch cultivation**. FC RLU: Fold change of relative luminescence units; PC: Principal component of the respective analysis. (a) and (b): given are means of measurements of three experiments (i.e. n = 3) ± SD; (3): each dot represents one ICM MS measurement. Dashed lines illustrate at which point culture alteration is detectable with the respective method.

Cell viabilities as assessed by trypan blue remained constant over 120 h of batch cultures. A first drop in cell viability was noticed between 120 and 144 h (Figure 1 a).

In ICM MS analysis, a total of approx. 160 *m/z *values was monitored in a mass to charge (*m/z*) range of 4,000 to 30,000. Principle component analysis (PCA; Figure 1 c) of ICM MS results showed no clear group discrimination during the first 96 h of cultivation. Interestingly, cell samples obtained from 120 h of cultivation onwards appear as distinct groups in PCA analysis.

The concentration of the monitored apoptosis marker (caspase-9 activity; Figure 1 b) began to increase between 96 and 120 h, i.e. concomitantly with PCA analysis (Figure 1).

As a result, ICM MS as reported here allowed for rapid detection of cell viability changes approx. 24 h earlier than standard culture monitoring and concomitant with the detection of an early, not "at-line" applicable apoptosis marker.

Closer data analysis allowed the identification of an apoptosis related subset of m/z values. Using the software ClinProTools (CPT; Bruker Daltonik) it was possible to develop a classification model which points toward classification of unknown samples regarding their viability/apoptosis state (Table 1). The classification power was illustrated as positive predictive value (PPV) which is the number of correctly classified samples over the total number of classified samples. All biological samples were analyzed as 6-8 technical replicates, meaning in theory a PPV > 50% is sufficient for classification.

**Table 1:** Details of classifying "unknown" samples using the CPT model

"unknown" sample [h]	Drop of viability [Y/N]	Apoptosis detection [Y/N]	Class	PPV [%]
**48**	N	N	Viable (no apoptosis signal)	94
**72**	N	N		
**96**	N	N		

**120**	N	Y	Early apoptotic	83

**144**	Y	Y	Late apoptotic	100
**192**	Y	Y		
**240**	Y	Y		

## Conclusion

We introduced a fast and robust ICM MS method for predictive cell culture monitoring. Viability changes can be detected up to 24 h earlier compared to standard methods (e.g. trypan blue).

We identified a specific MS signature (condensed subset of original spectra) of *m/z *values related to cell stress and apoptosis.

A model built on the basis of this signature allows classification of unknown samples regarding their viability/apoptosis level.

These results will be substantiated by assessment of further cell lines as well as monitoring attributes other than cell stress/apoptosis (e.g. product titer or metabolite progression).
